# Opportunities and challenges of radiofrequency ablation for substernal goiter: a case report

**DOI:** 10.3389/fonc.2025.1651824

**Published:** 2025-08-13

**Authors:** Zhiming Han, Lei Feng, Nan Wang

**Affiliations:** 1 Department of Ultrasound Medicine, Gansu Provincial Hospital, Lanzhou, China; 2 First Clinical Medical School, Gansu University of Chinese Medicine, Lanzhou, China

**Keywords:** substernal goiter, vascular dysplasia, radiofrequency ablation, intervention, efficacy

## Abstract

Substernal goiter (SG) refers to an enlargement of the thyroid gland that extends below the sternal notch or clavicle. While most cases are benign, a few may be malignant. Surgical resection is the standard treatment for SGs that cause clinical symptoms. This case report presents a 60-year-old female SG patient with a history of thyroidectomy and congenital vascular anomalies, who developed symptoms including significant airway compression and difficulty swallowing liquids. Following a detailed imaging and pathological evaluation, ultrasound-guided radiofrequency ablation was performed at the patient’s request. The procedure was successful and led to a favorable clinical outcome. This case highlights a potential alternative treatment approach for patients with SG, particularly those for whom surgery may pose additional risks.

## 1 Introduction

Substernal goiter (SG) was first described by Haller in 1794 and refers to an extension of the thyroid gland into the thoracic inlet. Although there has not yet been a uniform definition of SG in the literature, SG is commonly identified when thyroid tissue is observed below the sternal notch or clavicle on physical examination or imaging, typically with the patient in a supine position and the neck slightly extended. Its prevalence ranges from 5% to 20% (1–3). The pathogenesis of SG is not fully understood, which includes primary ectopic thyroid cells in the foregut endoderm and secondary metastases from the cervical thyroid to the mediastinum (4). The majority of SGs(Substernal goiters) are secondary, and it is noteworthy that the incidence of SG following thyroidectomy has been reported to range from 3% to 13% (5–7), as was the case in the patient described here, who developed SG after undergoing a right thyroid lobectomy.

Most SGs grow slowly and do not cause specific clinical manifestations. Only some of the larger SGs compress the trachea or esophagus, resulting in dyspnea, dysphagia, and hoarseness. Less commonly, SG may present with signs of hyperthyroidism or superior vena cava syndrome. Currently, Surgical resection remains the standard treatment for SGs that cause compressive symptoms, which includes transcervical incision approach resection and open thoracotomy (1). Although most SGs can be treated by surgical excision, some patients are still at risk for bleeding, damage to the laryngeal recurrent nerve, hoarseness, hypoparathyroidism, prolonged hospitalization, significant scarring, and other anesthetic complications (2). A meta-analysis by Charles et al. (5) reported a 4.41% overall complication rate for SG surgery, with specific issues including superior vena cava syndrome, tracheal collapse, and the need for tracheostomy. Despite these risks, few studies have explored non-surgical treatment options for SG, particularly for large goiters. Radiofrequency ablation (RFA), as a new treatment modality, has gained extensive clinical experience in the treatment of benign thyroid nodules (6–8).

In this case, we applied ultrasound-guided RFA to treat a substernal goiter in a patient with prior thyroid surgery and congenital vascular anomalies. The procedure was well tolerated and yielded favorable outcomes, with minimal complications such as bleeding, nerve injury, or postoperative pain. The patient also reported high satisfaction with the cosmetic results. In addition, we analyzed the technical points of radiofrequency ablation and evaluated its possibilities and challenges for the treatment of SG.

## 2 Case report

**Figure 1:**
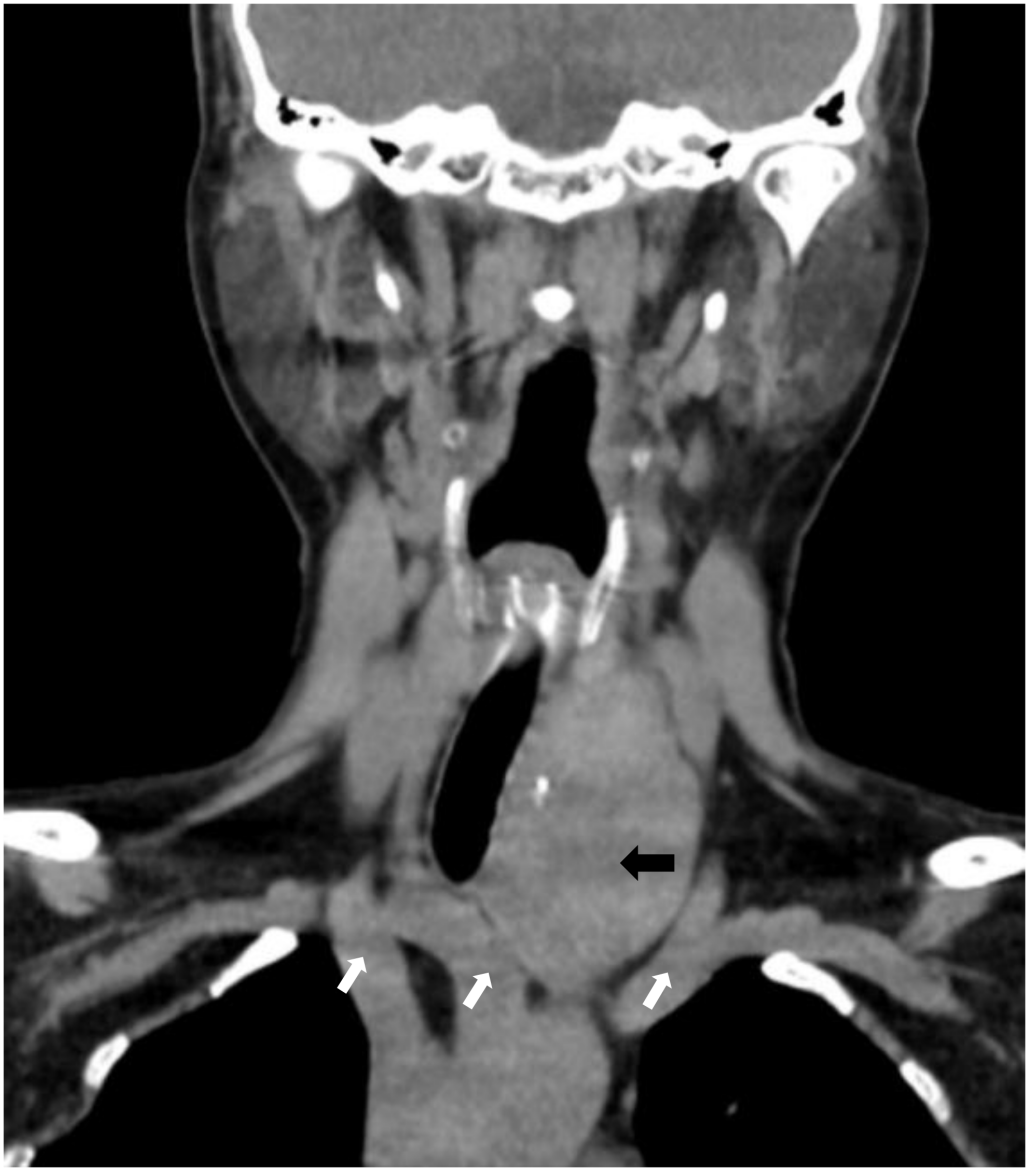
CT shows an enormous substernal goiter compressing the trachea. (black arrow). Right common carotid artery, right subclavian artery, left cephalic trunk. (image from left to right, white arrows).

**Figure 2:**
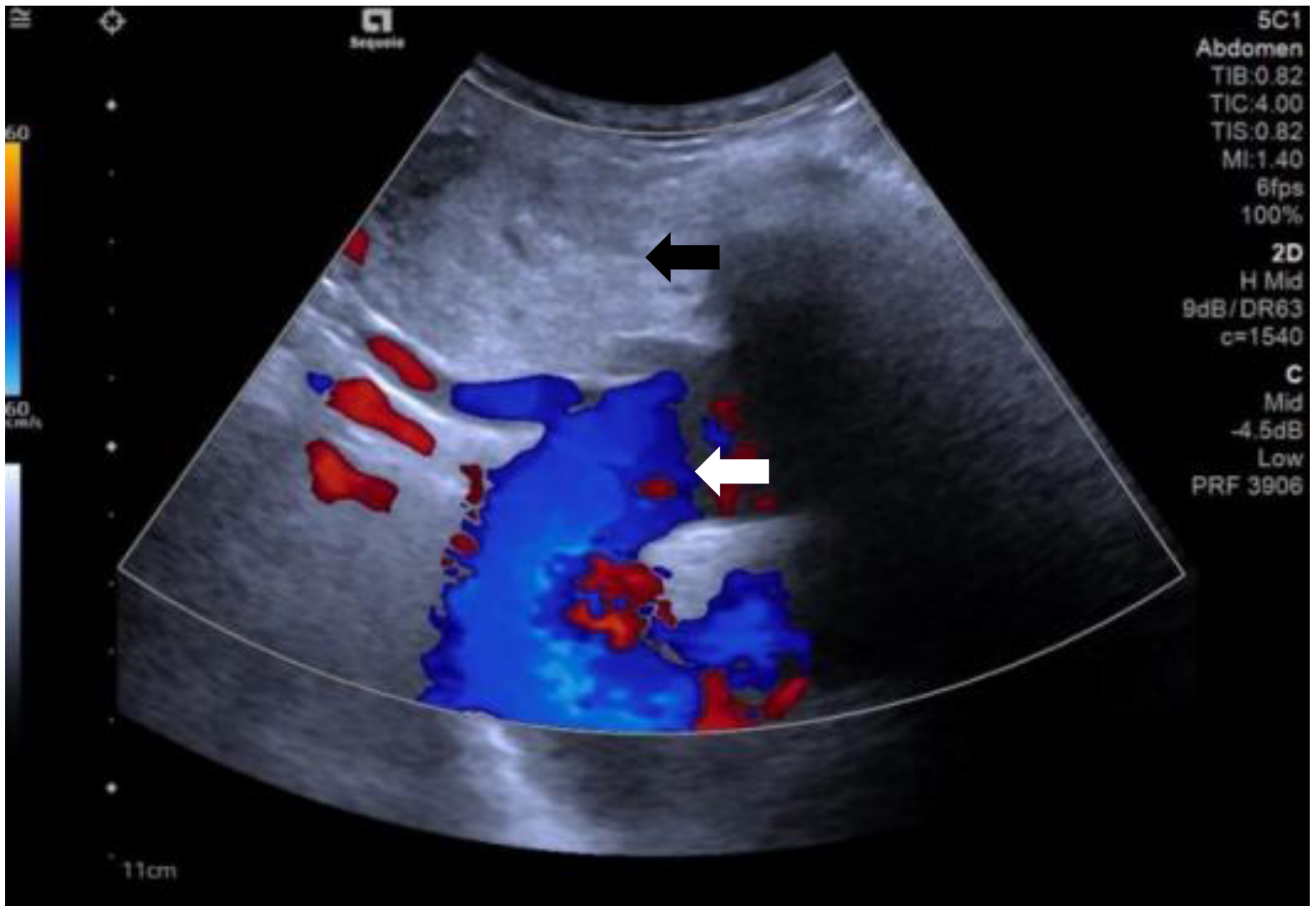
The ultrasound shows substernal goiter (black arrow) compressing the aortic arch and its branch vessels. (white arrow).

A 60-year-old female patient presented to our hospital with a history of thyroid nodules found for more than 20 years. Recently, she had developed airway compression and choking while drinking water. She had undergone a right thyroid lobectomy for benign thyroid nodules 5 years ago. After admission, CT examination showed a significant enlargement of the left thyroid lobe, with a mixed-density mass measuring approximately 4.6×4.2×4.5 cm. The lesion displaced the trachea to the right and extended into the anterior superior mediastinum, compressing the aortic arch, right subclavian artery, and left common carotid artery. Notably, the patient had a congenital vascular anomaly, with the aortic arch giving rise to the cephalic trunk, right subclavian artery, and right common carotid artery from left to right (**Figure 1**). The ultrasound examination showed that the left lobe of the thyroid gland was morphologically abnormal and significantly enlarged, with an inhomogeneous hypoechoic mass measuring about 4.9x4.5x4.7 cm, which was projecting into the upper mediastinum and compressing the aortic arch and its branch vessels (**Figure 2**). Laboratory tests for thyroid function and parathyroid function were normal, and a preliminary diagnosis of substernal goiter was made.

**Figure 3:**
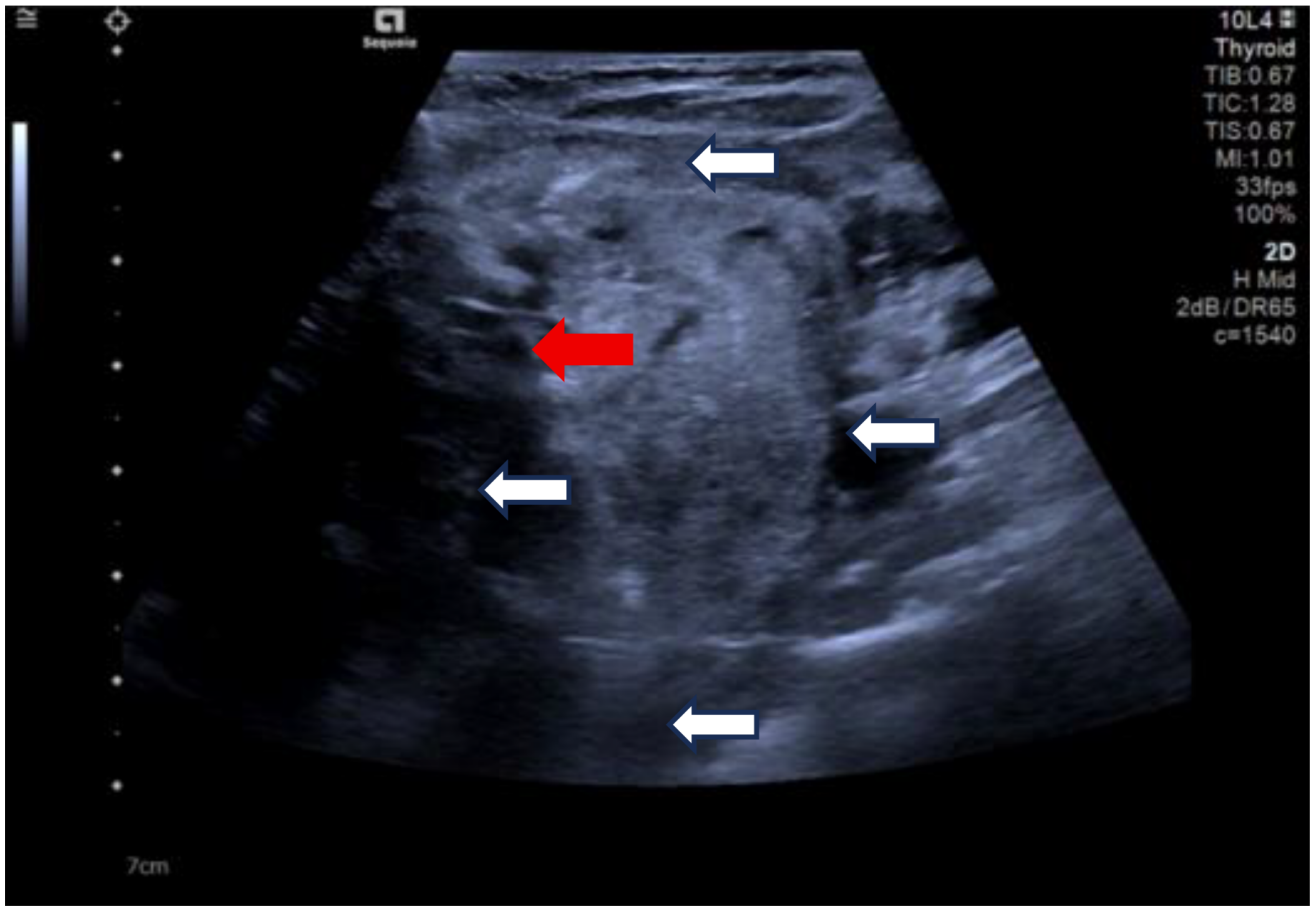
Clockwise from top, the anterior thyroid space, the lateral thyroid space, the posterior thyroid space and the paratracheal space are shown. (white arrows). Ablation needle position. (red arrow).

**Figure 4:**
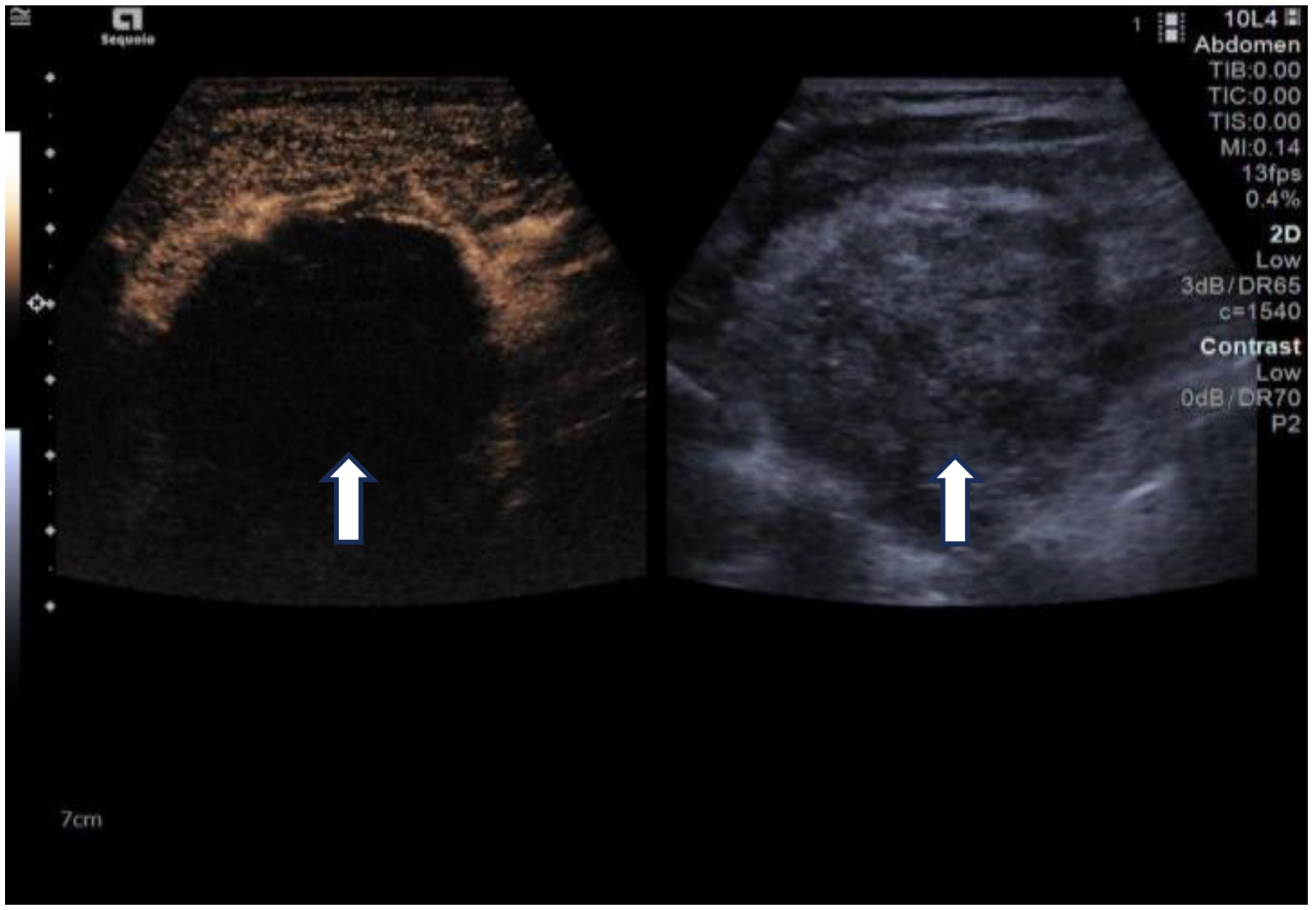
One-month follow-up after ablation. Left: Changes of the “black hole” on CEUS, Right: 2D ultrasound. (white arrows).

Due to her prior thyroid surgery and strong preference to avoid further surgery, the patient strongly requested a non-surgical treatment approach. After a thorough discussion, we performed an ultrasound-guided fine-needle aspiration biopsy (FNAB), which yielded a Bethesda III lesion and negative for BRAF gene. Based on the clinical and imaging examination, we opted for ultrasound-guided radiofrequency ablation (RFA) as the treatment modality. Before the ablation treatment, contrast-enhanced ultrasound (CEUS) showed that the nodule’s enhancement pattern was similar to the surrounding thyroid parenchyma, with synchronous enhancement and regression. After disinfecting the neck and spreading a towel, local infiltration anesthesia was applied, followed by injection of 30 ml of saline into the anterior and posterior spaces of the left thyroid and the paratracheal space under ultrasound guidance and real-time monitoring, and an isolation zone was formed around the giant nodule in the left lobe (**Figure 3**), and the thickness of the zone was ≥5 mm. Once an adequate isolation zone was established, the RFA needle (needle type: using an internally cooled 18G electrode, 10cm in length, and with a 10mm active tip size, powered by the RF generator (AJ-500A, Angel Medical))was inserted near the posterior aspect of the mass, and ablation commenced. The procedure was performed in a stepwise manner, retreating and adjusting the needle path to ensure complete treatment from the deep to the superficial portions of the nodule. The procedure lasted 20 minutes and 53 seconds at a power of 60W. While strong echogenicity on 2D ultrasound indicated near-complete ablation, residual contrast enhancement on CEUS suggested a small untreated area. Additional ablation was performed until both 2D ultrasound and CEUS confirmed complete lesion necrosis. The total ablation time was 25 minutes and 20 seconds. Throughout the procedure, the patient remained conscious and communicative, allowing continuous assessment of her vocal function. Vital signs, oxygen saturation, and ECG were monitored to ensure procedural safety. The patient experienced only mild neck discomfort and no complications such as bleeding or dyspnea. One month after the procedure, ultrasound of the patient’s follow-up showed a hypoechoic region at the ablation site with “black hole” changes on CEUS, indicating effective necrosis (**Figure 4**). Postoperative thyroid function remained normal, vocal function was preserved, and the patient expressed high satisfaction with the cosmetic outcome, especially the absence of a surgical scar.

## 3 Discussion

SG typically originates from the downward extension of the cervical thyroid into the mediastinum. This occurs because there is no significant anatomical barrier between the lower pole of the thyroid and the thoracic inlet, only a thin layer of connective tissue. Factors such as traction during swallowing, negative intrathoracic pressure during inspiration, and gravity can contribute to the thyroid’s descent along fascial planes into the mediastinum (9). The SG can be located either in the anterior or posterior mediastinum, though they are far more commonly found in the anterior mediastinum. Regardless of location, their blood supply is usually derived from the superior and inferior thyroid arteries (10).

RFA is a minimally invasive procedure that uses radiofrequency energy delivered through an electrode to generate an electric field within target tissue. Ionic agitation within the tissue, caused by the alternating current, produces frictional heat—a phenomenon known as the Joule effect—which leads to localized coagulative necrosis (8). Clinical studies have reported an overall complication rate of 2.38% and a major complication rate of 1.35% after RFA for thyroid nodules and thyroid cancer, including voice changes, nodule rupture, permanent hypothyroidism, and brachial plexus injury (11). In a large retrospective study, Che et al. compared outcomes between RFA (n=200) and surgical resection (n=200) for solid thyroid nodules. The RFA group demonstrated fewer postoperative complications, a higher rate of residual gland preservation, and a lower recurrence rate compared to the surgical group (6% vs. 1%, 11.9% vs. 2.9%, and 2.5% vs. 0.05%, respectively) (12). A literature search on PubMed using terms such as “Goiter,” “Substernal Goiter,” “Retrosternal Goiter,” and “Intrathoracic Goiter” revealed only one study by Cui et al., which explored the use of microwave ablation in 10 patients with substernal goiter. Their findings showed a mean volume reduction rate (VRR) of 66.7% at 3 months post-treatment, with no complications apart from mild intraoperative discomfort (13), which is consistent with our results. In contrast, thyroidectomy carries a notable risk of injury to the parathyroid glands, leading to hypoparathyroidism in up to 15% and hypocalcemia in up to 7.6% of cases (14, 15). The smaller size of the RFA needle allows for targeted ablation of nodules while preserving surrounding healthy thyroid and parathyroid tissue. This can minimize the risk of postoperative complications and reduce the need for lifelong hormone replacement therapy, thereby lowering the overall financial burden on patients (16, 17). Given these advantages, RFA presents a promising, less invasive alternative to surgery for selected patients with substernal goiter.

In cases of large SGs, particular attention must be given to venous congestion and compensatory thickening of arteries caused by chronic compression. In surgical management, a common strategy involves intraoperative identification and clamping of the affected vessels to minimize bleeding risk (1). In this case, the patient also had a combination of vascular dysplasia, which increased thermal injury during ablation. To address this, we injected sterile saline between the thyroid nodule and adjacent vascular structures to create a protective barrier, thereby reducing the likelihood of heat damage. The key anatomical spaces used for hydrodissection include: (1) anterior thyroid space: the space between the anterior thyroid peritoneum and the anterior cervical musculature (including the sternothyroid and thyroglossal muscles); (2) posterior thyroid space: the space between the posterior thyroid peritoneum and the long carotid muscles and carotid sheaths; (3) paratracheal space: the space between the medial thyroid peritoneum and the trachea, esophagus, and recurrent laryngeal nerve (18). The use of Color Doppler Flow Imaging (CDFI) during the procedure allows real-time visualization of blood vessels and continuous assessment of the adequacy of the isolation zone, further reducing the risk of vascular and nerve injury. Unlike surgery, which requires general anesthesia and endotracheal intubation, RFA is typically performed under local anesthesia. Intubation in surgical cases may be complicated by airway distortion from tracheal compression, placing additional demands on the anesthesiologist. Extubation must be managed carefully, as airway obstruction or wheezing may necessitate reintubation or emergency tracheostomy (1). In contrast, during RFA, airway patency and vocal cord function can be monitored in real-time through patient feedback and ultrasound imaging, reducing procedural risks. In this case, although the patient had a large nodule (4.6 × 4.2 × 4.5 cm), she exhibited no significant signs of respiratory distress preoperatively. This highlights the importance of thorough pre-procedural evaluation of respiratory function in determining whether RFA is a feasible and safe alternative to surgery in cases of SG.

Although the vast majority of SG can be removed through a cervical approach, Mussi believes that sternotomy should be used when the resected mass cannot be successfully removed from the incision or in recurrent goiters (19). Cohen identified four key risk factors that increase the likelihood of requiring sternotomy: (1) the presence of malignancy, (2) posterior mediastinal involvement, (3) goiter extending below the aortic arch (4) the presence of ectopic goiter (20). In a study by Ali et al., 4 out of 42 patients underwent sternotomy due to the size and extent of the goiter. One patient had a lesion extending below the aortic arch, two showed tracheal deviation and mediastinal masses on chest X-ray, and one had a goiter extending toward the tracheal bifurcation (21). In our case, the patient’s SG similarly compressed the trachea and reached the plane of the aortic arch after radiofrequency ablation treatment. Following RFA, her symptoms of neck compression improved significantly. It is also worth noting that the average duration of surgical resection for SG is approximately 106 minutes (range: 45–240 minutes). Specifically, transcervical procedures take an average of 101 minutes, while cases requiring sternotomy extend to 175 minutes on average (22). In contrast, the RFA procedure in our case was completed in under 30 minutes. Furthermore, because RFA requires only a small puncture site, it results in minimal or no visible scarring—an important consideration for patients with a prior thyroidectomy or those concerned about cosmetic outcomes. These advantages further underscore the potential of RFA as a viable alternative to surgery in selected cases of SG.

Despite the promising outcomes observed with radiofrequency ablation (RFA) in treating substernal goiter, certain factors may limit the effectiveness or feasibility of this approach. One major consideration is the anatomical location of the SG. While the majority of SGs (85–90%) are found in the anterior mediastinum, approximately 10–15% are located in the posterior mediastinum (10). The presence of the pericardium and sternum anterior to the posterior mediastinum can obstruct the path of the ablation needle. Moreover, deeper lesions are more challenging to visualize with ultrasound due to interference from bony structures and the trachea, increasing procedural risk. Computed tomography (CT) imaging plays a crucial role in preoperative planning by accurately defining the size, extent, and precise location of the goiter within the mediastinum. CT also aids in assessing whether a transthoracic surgical approach may be required, based on thyroid volume and invasion extent (22, 23). Another essential step before thermal ablation is obtaining a pathological diagnosis via biopsy. In previous guidelines (8), radiofrequency ablation was a commonly used treatment for benign thyroid nodules with a pathologic classification of Bethesda II. The pathology in this case was Bethesda III. However, the new Chinese multidisciplinary expert consensus suggests that exploratory ablation can be performed in patients with FNAC results of Bethesda class III or IV and BRAF-negative nodules, whose postoperative histologic results are mostly confirmed to be benign lesions, if it is not unethical or if the patient strongly requests non-surgical treatment (24). This case is also an exploratory attempt, so we hope that this case can provide a limited reference for the radiofrequency ablation treatment of such nodules.

## 4 Conclusion and patient perspective

This case demonstrates that RFA is a feasible and safe treatment option for substernal goiter, offering a minimally invasive approach with efficacy comparable to surgical resection. The main technical challenge lies in avoiding injury to blood vessels and nerves, underscoring the importance of thorough preoperative imaging and real-time intraoperative monitoring. While surgical resection remains the standard treatment for symptomatic benign substernal goiters, RFA presents a valuable alternative for patients with underlying cardiopulmonary conditions or those seeking to avoid visible scarring, particularly individuals with prior thyroid surgery. However, it should be noted that radiofrequency ablation cannot treat goiters or thyroid cancers that extend into the posterior mediastinum and are inaccessible to the radiofrequency needle (e.g., below the arch of the aorta).

## Data Availability

The original contributions presented in the study are included in the article/**Supplementary Material**. Further inquiries can be directed to the corresponding author.
