# Correction: Opportunities and challenges of radiofrequency ablation for substernal goiter: a case report

**DOI:** 10.3389/fonc.2025.1758451

**Published:** 2025-12-12

**Authors:** Zhiming Han, Lei Feng, Nan Wang

**Affiliations:** 1 Department of Ultrasound Medicine, Gansu Provincial Hospital, Lanzhou, China; 2 First Clinical Medical School, Gansu University of Chinese Medicine, Lanzhou, China

**Keywords:** substernal goiter, vascular dysplasia, radiofrequency ablation, intervention, efficacy

An incorrect number was provided for “The Gansu Youth Science and Technology Fund Program (2020-0406-jcc-0439)”. The correct number is “The Gansu Youth Science and Technology Fund Program (21JR1RA005)”.

The original version of this article has been updated.

